# Clinical Studies on the Diseases of the Eye

**Published:** 1885-08

**Authors:** 


**Clinical Studies on the Diseases of the Eye.** Including those of the Conjunctiva, Cornea, Sclerotic, Iris, and Ciliary Body. By Dr. FERDINAND RITTER VON ARLT, Professor of Ophthalmology in Vienna. Translated by LYMAN WARE, M. D. Price, $2.50. Philadelphia: P. Blakiston, Son & Co., No. 1012 Walnut street. 1885.

The accomplished author of the present work accedes to the wishes of his students, in furnishing a part of his clinical lectures for publication ; and he states that his object was, primarily, to give the physicians engaged in general practice a book of reference on diseases of the eye. It is distinct from the ordinary text-book, and contains the valuable experience of one of the ablest specialists of Germany. To those whose attention is, more or less, turned to ophthalmology, we commend this work as one of the most valuable contributions we have met.

